# Prevalence and Factors Associated with *Leishmania infantum* Infection of Dogs from an Urban Area of Brazil as Identified by Molecular Methods

**DOI:** 10.1371/journal.pntd.0001291

**Published:** 2011-08-16

**Authors:** Wendel Coura-Vital, Marcos José Marques, Vanja Maria Veloso, Bruno Mendes Roatt, Rodrigo Dian de Oliveira Aguiar-Soares, Levi Eduardo Soares Reis, Samuel Leôncio Braga, Maria Helena Franco Morais, Alexandre Barbosa Reis, Mariângela Carneiro

**Affiliations:** 1 Departamento de Parasitologia, Instituto de Ciências Biológicas, Universidade Federal de Minas Gerais, Belo Horizonte, Minas Gerais, Brazil; 2 Núcleo de Pesquisas em Ciências Biológicas, Instituto de Ciências Exatas e Biológicas, Universidade Federal de Ouro Preto, Ouro Preto, Minas Gerais, Brazil; 3 Departamento de Ciências Biológicas, Universidade Federal de Alfenas, Alfenas, Minas Gerais, Brazil; 4 Departamento de Farmácia, Escola de Farmácia, Universidade Federal de Ouro Preto, Ouro Preto, Minas Gerais, Brazil; 5 Gerência Regional de Controle de Zoonoses, Secretaria Municipal de Saúde, Prefeitura de Belo Horizonte, Belo Horizonte, Minas Gerais, Brazil; 6 Pós-Graduação em Infectologia e Medicina Tropical, Faculdade de Medicina, Universidade Federal de Minas Gerais, Belo Horizonte, Minas Gerais, Brazil; Institute of Tropical Medicine, Belgium

## Abstract

**Background:**

Various factors contribute to the urbanization of the visceral leishmaniasis (VL), including the difficulties of implementing control measures relating to the domestic reservoir. The aim of this study was to determine the prevalence of canine visceral leishmaniasis in an urban endemic area in Brazil and the factors associated with *Leishmania infantum* infection among seronegative and PCR-positive dogs.

**Methodology:**

A cross-sectional study was conducted in Belo Horizonte, Minas Gerais, Brazil. Blood samples were collected from 1,443 dogs. Serology was carried out by using two enzyme-linked immunosorbent assays (Biomanguinhos/FIOCRUZ/RJ and “in house”), and molecular methods were developed, including PCR-RFLP. To identify the factors associated with early stages of infection, only seronegative (n = 1,213) animals were evaluated. These animals were divided into two groups: PCR-positive (n = 296) and PCR-negative (n = 917) for *L. infantum* DNA. A comparison of these two groups of dogs taking into consideration the characteristics of the animals and their owners was performed. A mixed logistic regression model was used to identify factors associated with *L. infantum* infection.

**Principal Findings:**

Of the 1,443 dogs examined, 230 (15.9%) were seropositive in at least one ELISA, whereas PCR-RFLP revealed that 356 animals (24.7%) were positive for *L. infantum* DNA. Results indicated that the associated factors with infection were family income<twice the Brazilian minimum salary (OR 2.3; 95%CI 1.4–3.8), knowledge of the owner regarding the vector (OR 1.9; 95%CI 1.1–3.4), the dog staying predominantly in the backyard (OR 2.2; 95%CI 1.1–4.1), and a lack of previous serological examination for VL (OR 1.5; 95%CI 1.1–2.3).

**Conclusions:**

PCR detected a high prevalence of *L. infantum* infection in dogs in an area under the Control Program of VL intervention. Socioeconomic variables, dog behavior and the knowledge of the owner regarding the vector were factors associated with canine visceral leishmaniasis (CVL). The absence of previous serological examination conducted by the control program was also associated with *L. infantum* infection. It is necessary to identify the risk factors associated with CVL to understand the expansion and urbanization of VL.

## Author Summary

Visceral leishmaniasis (VL) is a disease caused by the parasite *Leishmania infantum*, and dogs are the most important domestic reservoirs of the agent. During recent decades, VL has expanded to large Brazilian urban centers. In the present work, we have demonstrated by using molecular techniques that the rate of canine infection as detected by serology has been considerably underestimated. Two groups of seronegative dogs (infected and non-infected according to molecular methods) were further evaluated from data obtained through interviews with owners of the animals. The factors associated with *Leishmania* infection in dogs were a family income of less than two minimum salaries, the knowledge of the owner regarding the vector, the dog spending most of its time in the backyard and the dog never having had a previous serological examination. Awareness regarding the factors associated with canine infection will improve health services and the understanding of the disease's expansion in urban areas.

## Introduction

Human visceral leishmaniasis (HVL) constitutes a public health problem that affects millions of people throughout the world [1]. Over the past decade, there has been an average of 3379 cases of HVL per year in Brazil, with an incidence of 1.9 cases per 100,000 inhabitants [2]. During this period, however, an increase in the prevalence of the disease has been observed in several urban areas, and this phenomenon may be attributed to high population density, increased migration, environmental changes, inadequate living conditions and vector adaptation [1], [3].

In South America and Europe, the causative agent of HVL is *Leishmania (Leishmania) infantum*, a protozoan parasite transmitted by sand flies of the Phlebotominae family, which are widely distributed in both wild and domestic surroundings [4]. Dogs are the main urban reservoirs and represent the major source of contagion for the vector by virtue of their high prevalence of infection and intense cutaneous parasitism [5]. Furthermore, it has been estimated that more than 50% of seropositive dogs are asymptomatic [6] and may remain free of clinical symptoms for several years or even throughout life [7].

The prevalence of canine visceral leishmaniasis (CVL) in endemic areas of Brazil ranges between 5.9 and 29.8% [8]–[13], although the serological methods employed in the detection of CVL exhibit low sensitivities and may underestimate the true value [14]–[15]. The Brazilian Ministry of Health, through the Control Program of Visceral Leishmaniasis (CPVL), has instituted specific measures to control the dissemination of the disease, and these include early diagnosis and treatment of human cases, identification and elimination of seropositive infected dogs, control of insect vectors and health education [2]. To date, however, the actions of CPVL have had little impact, and this negative outcome has been ascribed to delays in detecting and eliminating infected dogs, the tendency to replace infected dogs by susceptible puppies, and the low sensitivity of the available serological methods [16]–[18].

Although serological techniques lack the sensitivity required to detect *Leishmania* in the initial stages of infection, polymerase chain reaction (PCR) based assays can disclose the presence of protozoan DNA very early on, even before seroconversion [19]–[20]. Epidemiological studies employing modern molecular techniques have revealed that the prevalence of CVL in endemic areas in Europe is far greater than serological methods had previously suggested [15], [21]–[22]. According to De Andrade et al. [14], it is possible that as many as 62% of Brazilian dogs showing negative serological and parasitological tests for *L. infantum* would be classified as CVL-positive according to PCR and restriction fragment length polymorphism (RFLP) assays. A cohort study conducted by Oliva et al. [20] showed that most of the animals had PCR-positive results months before seroconversion. In addition, experimentally infected dogs have been found to be positive by conjunctival PCR by 45 days of infection [23].

To understand the expansion and urbanization of VL, it is necessary to identify the risk factors associated with human and/or canine infection. A number of publications have considered the factors influencing HVL [24]–[26], but the potential risk factors of the canine disease have received far less attention. Information concerning animal susceptibility and its association with race, size, type of hair and age is available [8], [27]–[28]. However, factors relating to the domiciliary and peridomiciliary environment, the socioeconomic status of the owner, the type of care provided for the animal, and specific animal behavior must be investigated to explain the importance of dogs in the maintenance of CVL in urban areas.

In view of the aforementioned problems an investigation was undertaken to look into the prevalence of *L. infantum* infection using PCR followed by RFLP and serological methods (ELISA). The factors associated with *L. infantum* infection among seronegative (determined by enzyme-linked immunosorbent assay - ELISA) and PCR-RFLP–positive dogs were also assessed. The *L. infantum* infection criterion proposed herein prioritizes CVL early onset. This study was conducted in Belo Horizonte, the capital of the State of Minas Gerais, located in Southeastern Brazil, which is considered an area of active transmission [29].

## Methods

### Ethical statement

The study was approved by the Committees of Ethics in Animal Experimentation of the Universidade Federal de Ouro Preto (protocol no. 083/2007), of the Universidade Federal de Minas Gerais (protocol no. 020/2007), and of the City Council of Belo Horizonte (protocol no. 001/2008). All procedures in this study were according to the guidelines set by the Brazilian Animal Experimental Collage (COBEA), Federal Law number 11794. Owners of the dogs participating in the project were informed of the research objectives and were required to sign the Informed Consent Form before sample and data collection.

### Study design

**Figure 1:**
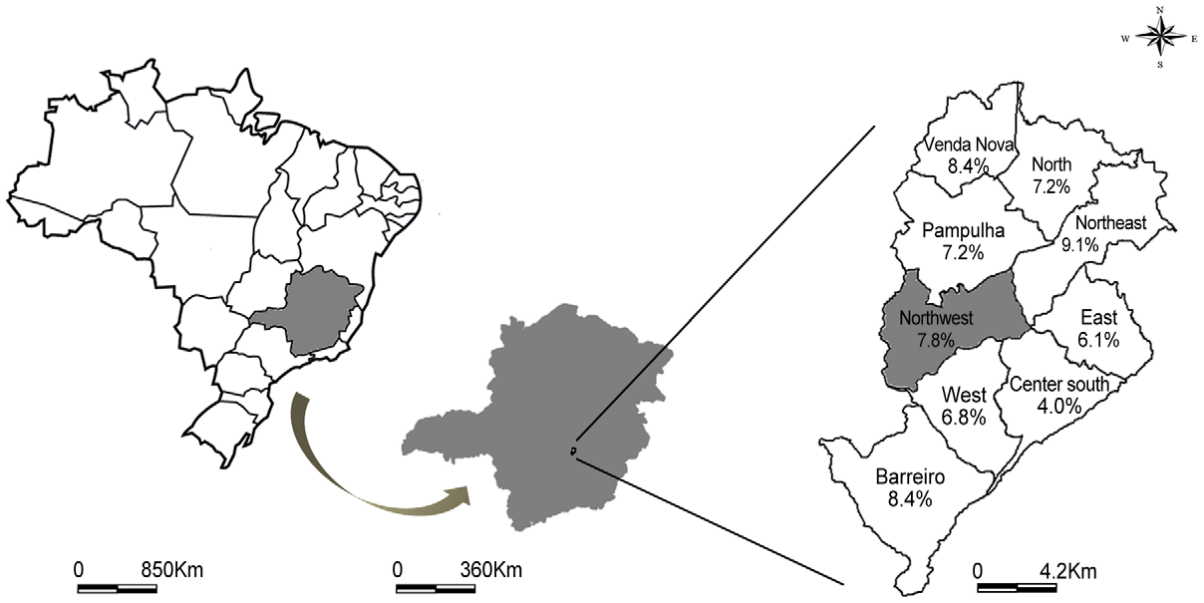
Municipality of Belo Horizonte, state of Minas Gerais, Brazil, subdivided into sanitary areas.

The cross-sectional study was conducted in 2008 in the northwest sanitary district of Belo Horizonte, which covers an area of 36.874 km^2^ (Fig. 1). According to the census by the Instituto Brasileiro de Geografia e Estatística in 2007, the human population of this area is 360,000. The canine population comprised 20,883 animals, according to the Zoonosis Control Management of the northwest sanitary district. At the time of the study, the prevalences of CVL in Belo Horizonte and its northwest sanitary district were 7.6 and 7.8%, respectively [30]. With an expected prevalence of CVL in the study area of between 5 and 10%, the 95% confidence interval, and an estimated precision of 1.5%, the appropriate sample size for the study was calculated to be approximately 1500 animals. Because of the high prevalence of seropositive dogs and the presence of human cases, the activities of the CPVL, including canine surveys (diagnosis and culling seropositive dogs), have been carried out in the study area annually. The present field work was done in close collaboration with the Municipality Health Service, and the data were collected during the canine survey census, conducted by the health agents, as part of CVLP's routine. The studied area was selected within the northwest sanitary district by convenience and was chosen because at that moment (2008) a canine survey was beginning in this area. The households visited by the CVLP in an area that comprised of 37 census tracts (according to the Brazilian Institute of Geography and Statistics) [31] were included in the present study. A total of 918 households were included in this study, and all dogs within selected houses were sampled.

### Collection of data

A trained research team interviewed the owners of the study animals using a previously tested, structured questionnaire that sought information regarding the following groups of variables: (i) knowledge about the disease (i.e., form of transmission and clinical signs of HVL); (ii) knowledge about the vector (characteristics and presence in the domicile and peridomicile); (iii) knowledge about the host (epidemiological importance of the host, clinical signs of leishmaniasis, and care of the dog); (iv) socioeconomic characteristics of the owner (per capita/family income, and schooling); (v) characteristics of the domicile, annexes and surroundings [i.e., structure of roof, floor and walls; number of rooms, including bedrooms; number of residents; presence of trees (particularly banana trees); rubble; exposed garbage; dead leaves; and vegetable garden]; (vi) method of garbage disposal (collected, burnt or buried); and (vii) presence of other domestic animals (birds, cats and cattle). The knowledge about the disease was validated according to self-reporting of the mainly symptoms of LVC and LVH. Vector recognition was acknowledged by self-reporting and validated by the showing of different diptera species samples (*Lutzomyia longipalpis* and *Aedes aegypti*) to the participants. The following information on the dogs was collected on an appropriate form: age, sex, size, hair type, breed, behavior (habits related to the place where the dog sleeps spends most of its time, *i.e.* in the street, in the residence, in the backyard), dog care, clinical examinations, past history of vaccination and serological exams previous to leishmaniasis. Some characteristics were defined by the health agents, such as breed, dog size, hair type and clinical evaluation. These characteristics are routinely obtained and registered in a standardized form used by CPVL in the canine survey. The hair type was defined according to the breed, i.e., collie was classified as long-furred hair, Doberman as short-furred. Dog size also was defined according to the breed, i.e., pinscher was categorized as small size, poodle as medium size and German shepherd as large size. According to the absence/presence of clinical infection signs, the dogs were categorized as asymptomatic, with no signs suggestive of disease, and symptomatic, with characteristic clinical signs of visceral leishmaniasis, such as opaque bristles, severe loss of weight, onychogryphosis, cutaneous lesions, apathy and keratoconjunctivitis.

### Collection of blood samples

A sample of peripheral blood (5 mL) was collected by puncture of the brachiocephalic vein and an aliquot transferred to a glass vial containing sufficient anticoagulant (ethylenediaminetetraacetic acid; EDTA) to give a final concentration of 1 mg/mL. The blood sample was centrifuged (1500–1800×*g*; 20 min), the buffy coat containing the leukocytes removed, resuspended in 10 mM Tris-HCl buffer (supplemented with 1 mM EDTA) in the proportion of 1∶1, and stored at −80°C until required for PCR-RFLP. The remaining portion of the blood sample was transferred to two separate filter papers for subsequent analysis by enzyme-linked immunosorbent assay (ELISA).

### Enzyme-linked immunosorbent assay

ELISA was performed in parallel in the Laboratory of Immunopathology of Universidade Federal de Ouro Preto (LIMP) and the Laboratory of Zoonosis of the Prefeitura Municipal de Belo Horizonte (LZOON). The presence of IgG against *Leishmania* in blood samples was determined using an “in-house” ELISA procedure performed at the LIMP. Soluble *Leishmania chagasi* (MHOM/BR/1070/BH46) antigen (SLA) was prepared by the method of Reis et al. [32] from promastigotes harvested from stationary-phase liver infusion tryptose cultures. The concentration of protein in the SLA solution was determined as previously described [33] and adjusted to 1000 µg/mL. Diluted SLA was divided into small portions and stored at −70°C until required for assay.

In the ELISA procedure, 96-well MaxiSorp™ microplates (Nalge Nunc Int., Rochester, NY, USA) were coated with SLA (2 µg/well) and maintained overnight at 4–8°C. Wells were then washed, and eluates from blood dried on filter paper were added at 1∶80 dilution. To perform the reaction, filter paper was thawed and 5-µm-diameter spots eluted in casein-PBS for testing by ELISA. The wells were washed again prior to the addition of peroxidase-conjugated sheep anti-dog IgG (anti-heavy chain specific; Bethyl Laboratories Inc., Montgomery, TX, USA). After further washes, chromogenic substrate (*O*-phenylenediamine; Sigma–Aldrich, St. Louis, MO, USA) was added, and the absorbance was read on an automatic EL 800G ELISA microplate reader (Bio Tek Instruments, Winooski, VT, USA) at 492 nm. The anti-IgG conjugate concentration employed (1∶16,000 dilution) was determined by a block titration method employing positive and negative standard sera. The cut-off value was established as the mean absorbance value +2 SD from 20 eluates from blood of uninfected dogs dried on filter paper.

Duplicate filter papers were submitted to ELISA at LZOON using a kit developed by Fundação Oswaldo Cruz, *EIE – Ensaio Imunoenzimático para diagnostico da leishmaniose visceral canina Bio-Manguinhos* (Rio de Janeiro, RJ, Brazil) and applied according to the supplier's instructions.

### Molecular methods (PCR-RFLP)

DNA was extracted from buffy coat fractions using Wizard™ Genomic DNA purification kits (Promega, Madison, WI, USA) according to the manufacturer's instructions. The primers used to amplify the conserved region of the *Leishmania* kDNA minicircle were as follows: forward: 5′-GGG (G/T)AG GGG CGT TCT (G/C)CG AA-3′; reverse: 5′-(G/C)(G/C)(G/C) (A/T)CT AT(A/T) TTA CAC CAA CCC C-3′ [34]. The reaction mixture consisted of 1× buffer [10 mM Tris-HCl, 50 mM KCl (pH 8.8)], 1.5 mM MgCl_2_, 2.0 µM dNTP, 1.0 pmol of each primer, 0.76 U of *Taq* polymerase (Sinapse, São Paulo, SP, Brazil), 2.5 µL DNA and Milli Q water to a final volume of 12.5 µL/well (MicroAmp® Fast Optical 96-Wells, Applied Biosystems, Foster City, CA, USA). PCR reactions were performed in a 96-well Verit Thermal Cycler (Applied Biosystems) using the following program: initial denaturation at 94°C for 1 min, followed by 40 cycles of 30 s at 93°C, 1 min at 64°C and 1 min at 72°C, with a final extension at 72°C for 7 min. DNA from *L. chagasi* (strain MHOM/BR/1972/BH46), obtained from the DNA reference library at LIMP, was used as positive control, while DNA from non-infected dogs, raised in the experimental kennels at UFOP, was used as negative control.

PCR amplicons (5 µL) were digested for 3 h at 37°C in 1 U of *Hae III* (Invitrogen, Carlsbad, CA, USA) in 1× buffer [10 mM Tris-HCl, 10 mM MgCl_2_ (pH 7.5)] and Milli Q water to a final volume of 15.0 µL/well (MicroAmp® Fast Optical 96-Well, Applied Biosystems) [35]. Restriction fragments, together with a 25 bp DNA ladder (Invitrogen), were electrophoresed in 10% polyacrylamide gels at 40 mA in 89 mM Tris base (pH 8.0), 89 mM boric acid and 2 mM EDTA. Bands were detected by silver staining, and the patterns were compared with those obtained using DNA from *L.* (*L.*) *amazonensis* (MHOM/BR/1973/M2269), *L.* (*Viannia*) *braziliensis* (MHOM/BR/1975/M2903) and *L.* (*L.*) *chagasi* (MHOM/BR/1972/BH46) from the DNA reference library at LIMP. Samples with very faint bands in PCR were extracted again and assayed by PCR to obtain better bands in the RFLP profile. All samples that did not show similar profiles to *L. infantum* DNA were excluded from the present study.

### Animal groups

Dogs were classified as seronegative if ELISA results were negative in both laboratories (LIMP and LZOON). The seronegative animals were categorized as (i) infected group: animals presenting positive PCR-RFLP for *L. infantum*; and (ii) non-infected group; animals presenting negative PCR-RFLP for *L. infantum*. These two groups were analyzed to identify factors associated with infection.

### Statistical analysis

Databases were generated using EpiData version 3.2 (EpiData Association, Odense, Denmark) by double entry of the results, and they were subsequently corrected, compared and analyzed using STATA version 11.0 software (Stata Corp., College Station, TX, USA).

To investigate the factors potentially associated with *L. infantum* infection, the infected and non-infected groups of animals were compared. A mixed logistic regression model [36] was employed to evaluate the association between the independent and dependent variables. This model was chosen on the basis that the sampling process included all dogs within a studied household, and it incorporated the underlying assumption that observations obtained from dogs in the same household were mutually dependent while observations from dogs in different households were independent. The xtmlogit function provide by Stata was used to perform the analysis and the household was included as a random effect.

**Figure 2:**
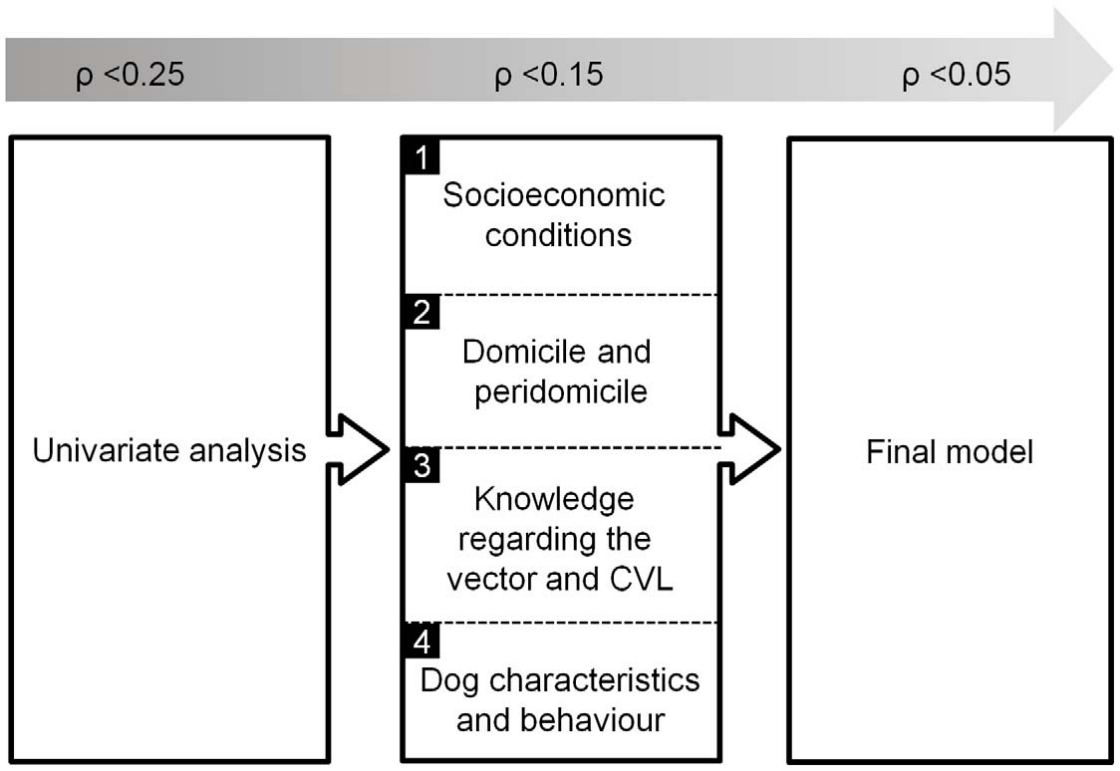
Hierarchical framework of risk factors for *Leishmania infantum* infection in dogs.

Univariate analysis using the mixed logistic regression model was conducted for all variables collected, and those that attained a p value<0.25 were included in the multivariate models. Hierarchical analysis levels were established on the basis of a hypothetical canine infection model that took into account the collected variables. The inclusion of variables in the model was based on a conceptual framework describing the hierarchical relationships between risk factors and canine *L. infantum* infection [37]. The variables were grouped in four levels: socioeconomic conditions; household and outside-home conditions; knowledge of vector and host; and dog characteristics and behavior (Fig. 2). Variables with a significance of p<0.15 in each hierarchical level were maintained in the next level. Variables presenting statistical significance at each level but with either collinearity or low frequency were excluded from the multivariate analysis, while categorical variables were transformed into dummy variables. Backward analyses were used to construct intermediate and final models, and likelihood ratio tests were used to adjust these models. Variables with significance levels of p<0.05 were maintained in the final model.

## Results

### Serological and molecular diagnosis of the study animals

Of the 1443 dogs studied, 230 (15.9%, 95% CI 14.1–17.9) were seropositive according to at least one ELISA. The results in each laboratory were 12% (LIMP) and 9.4% (LZOON). PCR-RFLP analyses revealed that 356 (24.7%; 95% CI 22.5–26.0) of the dogs studied were positive for *L. infantum* DNA. Only three showed molecular bands similar to *L. braziliensis*, and they were not included in the present study.

Among 1087 PCR-negative and 356 PCR-positive animals, 170 dogs (15.6%) and 60 dogs (16.8%), respectively, were seropositive in at least one ELISA. To investigate factors associated with *L. infantum* infection, those animals that were positive in at least one ELISA test (n = 230) were excluded. Therefore, among the 1.213 seronegative dogs, two groups were set up, according to PCR-RFLP: 296 (24.4%) positive and 917 (75.6%) negative.

### Characteristics of the dogs

Within the group of 1213 dogs included in the evaluation of associated factors, female (53.7%), medium-sized (52.3%) and short-haired (54.7%) animals predominated. The mean age was 54.2 months (SD 39.8), and the median was 48 months (IQR 24; 84). Most of the dogs (58.9%) had received a check-up by a veterinarian. The majority of the animals (97.6%) were asymptomatic (no signs suggestive of disease), and most (52.6%) had been acquired within the neighborhood of the owner's residence. Generally, the animals lived and slept in the backyard (83.7 and 77.7%, respectively), rather than inside the residence.

### Knowledge of the owners about the disease

Of the 918 dog owners who were interviewed, 903 (98.4%) had some knowledge about leishmaniasis, and of these, 533 (59.0%) knew about the forms of transmission. However, despite this rather widespread awareness, only 201 owners (21.9%) were familiar with the symptoms of HVL, and only 103 (11.2%) claimed to have knowledge of the vector of *Leishmania*. Around 4% of owners had seen the vector in their domicile and/or peridomicile. Concerning CVL, 328 owners (35.7%) stated that they were aware of the importance of dogs in the transmission of leishmaniasis, and 417 (45.4%) declared that they knew the symptoms of the disease in the dog. When asked about their views if their pet were found to be infected with *Leishmania*, 75.1% of owners stated that they would authorize euthanasia. Interestingly, of the 209 owners (22.8%) who had dogs with CVL in the past, 162 (77.5%) consented to euthanasia of their animal, whereas 37 (17.7%) sought treatment for their pet. At least one case of CVL had been recorded in the vicinity (same block) of many (49.5%) of the residences evaluated (data not shown).

### Housing conditions

A total of 918 households were selected. They had a mean of 1.57 (SD 1.17) dogs per household (1–9 dogs/house) and median of 1 (IQR 1; 2). The majority of the dwellings (563; 61.3%) were detached houses, while 873 (95.1%) had plastered walls, 754 (82.1%) had a garden and 909 (99.0%) were served by main sewage. Garbage was collected three or more times per week from 860 (93.7%) residences. The mean numbers (SD) of rooms and bedrooms per house were 7.2 (SD 2.8) and 2.7 (SD 1.0), respectively. Each dwelling had an average of 3.8 (SD 1.8) residents.

### Risk factors associated with *L. infantum* infection

A comparison between the infected (*n* = 296) and non-infected (*n* = 917) groups of animals was performed by multivariate analysis using the variables obtained from the interviews with owners and the records of individual dogs. The results of preliminary selection of the variables from the univariate analysis (p<0.25) are shown in Tables 1 and 2. The variables selected to build the final model (p<0.15) were knowledge of the owner regarding the vector (yes/no), knowledge of bite from the vector (yes/no), house treated with insecticide (no/yes), family income (<2 minimum salary/2–3 minimum salary/> 3 minimum salary), type of floor in the residence (other materials/tiles or wood), type of neighborhood (houses/houses with garden/lands), origin of dog (another district/present neighborhood), dog stays predominantly in the backyard (yes/no), where the dog sleeps (indoors/outdoors), and lack of previous CVL serological examination (no/yes).

**Table 1:** Distribution of seronegative dogs (*n* = 1213) according to the characteristics of the animals, Brazil 2008.

Variable	PCR-RFLP		Univariate analysisOdds Ratio(95% CI)	ρ values
	Positive*n* (%)	Negative*n* (%)		
Hair				
Short	173 (58.5)	491 (53.5)		
Long	123 (41.5)	426 (46.5)	1.2 (0.9–1.7)	0.17
Veterinary check ups				
Yes	151 (55.3)	527 (59.4)		
No	122 (44.7)	360 (40.6)	0.8 (0.6–1.1)	0.25
Symptomatic				
Yes	4 (1.4)	25 (2.7)		
No	292 (98.6)	890 (97.3)	0.4 (0.1–1.5)	0.20
Origin of the animal				
District of residence	154 (56.2)	459 (51.6)		
Other district	120 (43.8)	431 (48.4)	0.8 (0.6–1.1)	0.16
Dog staying predominantly in the backyard				
No	36 (13.1)	153 (17.2)		
Yes	238 (86.9)	737 (82.8)	1.4 (0.9–2.3)	0.10
Sleeping place				
Inside the house	51 (18.6)	208 (23.4)		
In the garden	223 (81.4)	682 (76.6)	1.4 (1.0–2.1)	0.08
CVL sorological examination previously				
Yes	183 (68.8)	662 (76.1)		
No	83 (31.2)	208 (23.9)	1.5 (1.1–2.2)	0.02
Age				
≤24 months	108 (36.5)	292 (31.8)		
>24 and ≤84 months	116 (39.2)	411 (44.8)	0.7 (0.5–1.0)	0.08
>84 months	72 (24.3)	214 (23.3)	0.9 (0.6–1.3)	0.59

**Table 2:** Distribution of owners (*n* = 918) of seronegative dogs according to the socioeconomic and environmental conditions and understanding of the disease, Brazil 2008.

Variable	PCR-RFLP		Univariate analysisOdds Ratio (95%CI)	ρ values
	Positive (%)	Negative (%)		
**Socioeconomic conditions**				
Family income				
>3 minimum wages*	72 (54.1)	281 (65.8)		
1 to 3 minimum wages	24 (18.1)	88 (20.6)	0.9 (0.6–1.5)	0.78
<1 minimum wages	37 (27.8)	58 (13.6)	2.4 (1.5–3.9)	0.00
Schooling				
University	49 (24.5)	134 (21.2)		
Secondary School	70 (35.0)	278 (44.0)	0.6 (0.4–0.9)	0.03
Primary school	78 (39.0)	217 (34.3)	0.9 (0.6–1.4)	0.71
Illiterate	3 (1.5)	3 (0.5)	4.5 (0.8–25.9)	0.09
**Environmental conditions**				
House walls plastered				
Yes	174 (95.6)	594 (96.1)		
No	8 (4.4)	24 (3.9)	1.8 (0.9–3.9)	0.12
Floor construction				
Tiles/wood	144 (78.3)	521 (84.3)		
Other	40 (21.7)	97 (15.7)	1.4 (1.0–2.2)	0.07
Insecticide-sprayed house				
Yes	134 (73.6)	425 (69.7)		
No	48 (26.4)	185 (30.3)	1.4 (0.9–2.0)	0.10
Open litter				
Yes	39 (21.2)	123 (19.8)		
No	145 (78.8)	497 (80.2)	1.3 (0.9–1.9)	0.20
Neighbourhood				
Houses	51 (27.7)	131 (21.1)		
Houses with gardens	118 (64.1)	439 (70.8)	0.6 (0.4–0.9)	0.02
Land	15 (8.2)	50 (8.1)	0.6 (0.3–1.2)	0.14
**Understanding the disease**				
Regarding the vector				
Yes	24 (13.0)	68 (11.0)		
No	160 (87.0)	552 (89.0)	1.4 (0.9–2.2)	0,18
Regarding the reasons why dogs have to be eliminated				
Yes	150 (81.5)	462 (74.4)		
No	34 (18.5)	159 (25.6)	1.6 (1.0–2.4)	0.03
Owner arranged examination of dog				
Yes	132 (72.1)	488 (80.0)		
No	51 (27.9)	122 (20.0)	0.7 (0.5–1.1)	0.11

*Brazilian minimum wages (Brazilian monthly minimum wage = U$262).

Infection with *L. infantum* (as detected by PCR-RFLP) was associated with a family income of less than twice the minimum salary (OR 2.3; 95% CI 1.4–3.8), knowledge of the owner regarding the vector (OR 1.9; 95% CI 1.1–3.4), dog staying predominantly in the backward (OR 2.2; 95% CI 1.1–4.1) and lack of previous CVL serological examination (OR 1.5; 95% CI 1.1–2.3) (Table 3).

**Table 3:** Risk factors for *Leishmania infantum* infection in seronegative dogs according to hierarchical model, Brazil 2008.

Variable	Crude Odds Ratio(95% CI)	Adjusted Odds Ratio(95% CI)
Family income<2 wages *versus* >3 minimum wages*	2.4 (1.5–3.9)	2.3 (1.4–3.8)
Knowledge of the owner regarding the vectoryes *versus* no	1.4 (0.9–2.2)	1.9 (1.1–3.4)
Dog staying predominantly in the backyardyes *versus* no	1.4 (0.9–2.3)	2.2 (1.1–4.1)
CVL serological examination previouslyno *versus* yes	1.5 (1.1–2.2)	1.5 (1.1–2.3)

*Brazilian minimum wages (Brazilian monthly minimum wage = U$262).

## Discussion

The results in the present investigation show that the prevalence of *L. infantum* infection in dogs as determined by PCR-RFLP (24.7%) is higher than that detected by serology (15.9%). Such divergent values are highly significant because they demonstrate that the magnitude of CVL in this study area, which is under constant CPVL intervention, has been underestimated. Factors associated with early *L. infantum* infection (PCR-RFLP+) were the socioeconomic conditions of the owner, the behavior of the dog, knowledge of the owner regarding the vector and the care the dogs had received. These results are relevant because they allow better understanding of the transmission of VL in a large city such as Belo Horizonte where leishmaniasis is expanding [29], [38]. Moreover, the diagnosis of canine infection by *L. infantum* was achieved through the application of PCR-RFLP, which indicated the early onset of CVL. Additionally, as the data originated directly from dog owners and their respective animals, it was possible to perform a detailed analysis of a range of information and to determine the factors associated with CVL.

Studies in European endemic areas have also demonstrated an elevated prevalence of infection (typically 60–80%) by PCR in comparison with that indicated by serology (generally<30%) [15], [39]. Species identification was essential, especially because Belo Horizonte is an area of the simultaneous occurrence of cutaneous and visceral leishmaniasis and the dog can be host for both parasites [40]. Among the examined samples, only three showed molecular bands similar to *L. braziliensis*, and they were not included in the present study. Approximately a quarter of seronegative dogs were infected by *L. infantum* according to PCR-RFLP. These false-negative animals were likely within an “immunological window” that occurs prior to seroconversion, during which period B lymphocytes do not secrete polyclonal antibodies, and consequently, serological methods are less sensitive at this stage of the infection [41].

It is possible that false-negative dogs remain in the community as undisclosed reservoirs and, thus, interfere with the effectiveness of control measures. Indeed, despite recent intense efforts to eliminate seropositive dogs, no reduction in the incidence of HVL or CVL has been observed in urban areas [42]. Little is known if seronegative/PCR-positive dogs are immunologically resistant to *Leishmania* [43] or if they will develop the disease. However, it is possible to state that such animals have had previous contact with the parasite. Such information is relevant because canine positivity for *Leishmania* is included among the indicators for the prioritization of target control areas by the Ministry of Health. Although molecular biology methods are more promising in identifying infection, their use in the field requires further standardization and optimization.

HVL is favored by precarious socioeconomic and housing conditions, migratory movements and the presence of vector and reservoir in the domestic environment [24]–[26], [44]. However, little is known about the risk factors that facilitate *Leishmania* infection in the main reservoir of the disease, namely, the domestic dog. To obtain a better understanding of these factors, comparisons were made between non-infected (seronegative/PCR-RFLP negative) animals and those infected (seronegative and PCR-RFLP positive). The decision to use PCR-RFLP–positive and seronegative animals was due to the detection of *L. infantum* in the initial stage of infection before seroconversion [19]–[20].

Regarding socioeconomic conditions of the owner, animals belonging to families with incomes of less than twice the minimum salary were twice as likely to be infected in comparison with dogs of higher-income families (three minimum salaries). In this context, family income is a proxy variable of socioeconomic status and is probably associated with the structure of the most vulnerable domiciles. Indeed, Oliveira et al. [26] demonstrated an association between HVL and family income following a study in the metropolitan area of Belo Horizonte. These data are also consistent with literature confirming that VL is more frequent in areas of precarious socioeconomic status [45].

In general, dog owners showed little knowledge of phlebotomine sand flies. Interestingly, however, dogs whose owners knew about the vector were twofold more likely to acquire the infection than those whose owners were not familiar with the insect. This variable can be understood as an indirect measure of exposure to phlebotomines and shows the importance of using proxy. A similar observation has been reported by Moreno et al. [44], who noted that in the metropolitan area of Belo Horizonte, the likelihood of being infected by *Leishmania* is six times greater for people who have seen the vector than for those who have not. A high density of *Lu. longipalpis* was observed in the present study area [38], so it is not surprising that the most respondents had noted the presence of the vector in their residences and neighborhood.

Dogs that usually lived in the backyard were twice as likely to acquire the infection as those that remained inside the house. According to Galvez et al. [46], living outdoors is significantly associated with serological positivity for the parasite among canines. In the recent survey performed in Granada, Spain, dogs that slept outdoors were at greater risk than those sleeping indoors because of vector density [47]. On the other hand, Cabrera et al. [48] reported that the risk of infection by CVL is similar for dogs that live within the perimeter of a residence and those that wander the streets or woods.

To reduce the risk of CVL, some preventive measures may be adopted, including the maintenance of dogs in closed kennels during periods of intense vector activity, the reduction of microenvironmental factors that favor the development of the vector in the residence, and the use insecticide-impregnated collars [22], [49]. However, the implementation of such measures depends not only on the degree of awareness of the dog owner about the disease but, mainly, on socioeconomic issues, because the most affected population could not afford to leave their dogs in kennels or buy impregnated collars. Only 35.5% of owners knew of the important role of dogs in the transmission of *Leishmania*, and 45.5% had knowledge of the symptoms of CVL, although 22.8% reported previous ownership of a dog that had contracted CVL.

Animals serologically tested by the CPVL previously were less likely to be infected. This finding indicates that seropositive dogs have been removed regularly by the control measures and that dogs that remain seronegative in successive tests are more likely to be CVL-free. Unfortunately, however, the replacement of dogs within the study area is frequent, and these animals would be more susceptible to infection by *L. infantum* [50]. The mean age of infected dogs was 49.8 (SD 41.3) months, whereas the mean age of non-infected dogs was 54.5 (SD 39.0) months. One possible explanation for this result is that the CPVL had removed seropositive dogs during the canine survey. Therefore, PCR was detecting *L. infantum* infection early, in younger dogs. Although the univariate analysis was significant, dog age was not associated with *L. infantum* infection. Galvez et al.[46] examined the age at which seroprevalence showed a bimodal distribution, with one peak appearing in the young dogs (1–2 years) and a second, more evident, peak among the older dogs (7–8 years). On the other hand, França-Silva et al. [8] observed that the prevalence of infection was not correlated with dog age.

The emergence of leishmaniasis in Belo Horizonte dates from the late 1980s, when the disease spread from areas marked by poor socioeconomic conditions [51]. At the present time, the disease is increasing, and VL has been detected in all regions of the city [38]. Indeed, the urbanization of VL is a current reality in many Brazilian cities.

We tried to identify domiciles that were most vulnerable to the presence of the vector and occurrence of infection. However, no variable related to households was maintained in the final model. In a study conducted in Northeastern Brazil, the risk of HVL was greater in residences that lacked sewage services and garbage collection [25]. In the present study, no influence of such factors on the prevalence of CVL was found, as 99% of domiciles were served by a main sewage connection and nearly all received garbage collection.

Even though our sampling procedure was not probabilistic, the studied households were sampled from a census survey, and the investigated blocks are representative of the northwest sanitary district. This study was not designed to evaluate a representative sample of Belo Horizonte but to assess the prevalence of infection by PCR-RFLP in seronegative dogs and identify risk factors for infection in these animals. However, the northwest sanitary district is representative of the city, with buildings, commerce, residences and green areas. Moreover, the main limitation of a cross-sectional study in identifying risk factors is that it does not permit causal inferences because time factors were not evaluated.

Although it is not easy to attribute the associated factors with new measures that can be adopted by CPVL, it is necessary to better investigate the factors associated with VL expansion in urban areas. Improved understanding of urbanization processes in large cites such Belo Horizonte can help the CPVL to adopt measures that are more effective at controlling the spread of the disease. It is important to emphasize that the control of HVL depends on the management of CVL because dogs are the main urban reservoir of *Leishmania* and represent the main source of phlebotomine infection.

The Control Program in Brazil used ELISA for screening and IFAT as a confirmatory test to identify seropositive dogs which are them euthanized. Due to the low level of humoral immune response, some of the infected dogs by *L. infantum* could not be detected. Therefore, using only seronegative dogs, this paper focuses on those animals that are positive by PCR and are not identified by the control program. Considering that the currently available serologic methods lack sufficient sensitivity and/or specificity to accurately identify all infected dogs, the employment of molecular diagnosis to detect the CVL infection before antibody production could be an efficient alternative. This study showed for the first time the identification of factors associated with early stage of CVL in animals seronegative with PCR-positive for *L. infantum* and therefore could contribute to better understanding of the involvement of this reservoir in urban-VL epidemiology. Additionally, for better investigation of the factors associated with VL expansion in urban areas further studies are required using a cohort study approach.
